# Effect of acupuncture for methadone maintenance treatment patients: study protocol of a randomized clinical trial

**DOI:** 10.1186/s13063-020-04930-x

**Published:** 2020-12-07

**Authors:** Hao Wen, Shichao Xu, Jingchun Zeng, Shuqi Ge, Yuan Liao, Chunzhi Tang, Songhua Xiao, Liming Lu

**Affiliations:** 1 Department of Neurology, Sun Yat-sen Memorial Hospital, Sun Yat-sen University, 107 Yanjiang West Road, Guangzhou, 510120 People’s Republic of China; 2 The Affiliated Brain Hospital of Guangzhou Medical University (Guangzhou Huiai Hospital), Guangzhou, 510006 People’s Republic of China; 3 The First Affiliated Hospital of Guangzhou University of Chinese Medicine, Guangzhou, 510405 People’s Republic of China; 4 Medical College of Acu-Moxi and Rehabilitation, Guangzhou University of Chinese Medicine, Guangzhou, China

**Keywords:** Opioid dependence, Jin’s three-needle technique, Acupuncture, Methadone maintenance therapy

## Abstract

**Background:**

Opioid dependence is an increasing public health problem all over the world. Patients with opioid dependence have to receive methadone maintenance therapy (MMT) as replacement therapy for years or even for their entire life. Acupuncture as a kind of therapy has been used to treat substance dependence for many years. Jin’s three-needle acupuncture (JTN), a type of acupuncture technique, has been applied to treat various diseases for several decades. However, JTN as an acupuncture technique has not been used to treat patients receiving MMT. Therefore, we designed a randomized controlled trial to evaluate the efficacy and safety of acupuncture as adjunctive therapy for patients receiving MMT.

**Methods/design:**

This study is a parallel-arm, randomized controlled trial that aims to evaluate the efficacy and safety of acupuncture as adjunctive therapy for patients receiving MMT. A total of 140 eligible participants who range in age from 18 to 60 years and fulfil the Diagnostic and Statistical Manual of Mental Disorders, 5th edition (DSM-V), for opiate dependence will be enrolled into this study. All eligible participants will be randomly assigned to the acupuncture group or routine group in a 1:1 allocation ratio. Participants who are enrolled in the acupuncture group will receive MMT and JTN treatment for 30 min per session. Meanwhile, those who are assigned to the routine arm will receive MMT only. All 18 sessions of JTN treatment will be delivered over 6 weeks (3 per week) and followed by a 4-week follow-up period. The primary outcome measure will be the visual analogue scale (VAS) for drug craving and the daily consumption of methadone (DCOM). Secondary outcome measures will include the urine test for opioid use, the 36-item Short Form Survey (SF-36), the Beck Anxiety Inventory (BAI), the Beck Depression Inventory II (BDI-II) and Pittsburgh sleep quality index (PSQI). VAS, DCOM, BAI, BDI-II and the urine test for opioid use will be evaluated at baseline, the second week, the fourth week, the sixth week and the tenth week. SF-36 and PSQI will be assessed at baseline, the fourth week, the sixth week and the tenth week.

**Discussion:**

The results of this trial will provide evidence on the efficacy and safety of acupuncture as adjunctive therapy for patients receiving MMT.

**Trial registration:**

Chinese Clinical Trial Registry ChiCTR1900026357. Registered on 2 October 2019.

## Background

According to the World Drug Report 2018, approximately 31 million people are affected by opioid use disorders through the world, which cause the greatest burden of severe disease and drug-related deaths worldwide [1]. In China, there are approximately 2.5 million people using illicit drugs, of which opioids and methamphetamines represent the majority [2].

Opioid dependence is a chronic psychiatric disorder. Discontinuation of the drug abruptly induces specific physiological and psychological changes called withdrawal, which is a temporary stage opposing intoxication towards re-establishment of the patient’s neuronal system. Withdrawal symptoms mainly include sweating, shaking and diarrhoea in the first few days, accompanied by dysphoria, depression, anxiety and insomnia, which may continue for several months [3, 4]. It is an unadaptable state associated with withdrawal symptoms and severe craving, which may lead to the high risk of relapse. The primary goal of treatment for opioid dependence usually focuses on achieving control of withdrawal symptoms and reducing cravings. Methadone has been used to treat withdrawal symptoms for several decades. Patients with opioid dependence have to receive MMT as replacement therapy for years or even their entire life. However, side effects of MMT still exist and have been reported to range widely, especially during the early weeks of methadone stabilization [5]. Patients often complain of insomnia and cravings during MMT, which contribute to the risk of relapse [6]. Therefore, it is necessary to seek a new treatment with efficacy and safety for patients receiving MMT.

Acupuncture has been applied to treat diseases in China for thousands of years, gaining popularity in Western countries as an alternative and complementary therapy [7]. In 1996, the World Health Organization accepted that acupuncture, as a kind of therapy, is appropriate for the treatment of drug use disorders [8]. Compared with drug treatment, acupuncture for opioid dependence has zero side effects and is convenient and inexpensive [9]. Acupuncture may inhibit the activation of specific brain regions related to drug craving, which support its potential as a therapy for drug craving [10]. Liang Yan reported that acupuncture may alleviate the depression and anxiety associated with heroin dependence [11]. Some basic studies have revealed that acupuncture might potentially reduce relapse through inhibiting attention bias to heroin [12], lowering complications of drug dependence [13] and modifying the morphine withdrawal syndrome [14]. To our knowledge, JTN, a type of acupuncture technique, has been applied to treat diseases for decades and has formed a school of acupuncture technique in South China. The concept of ‘mind’ means mental activities in traditional Chinese medicine (TCM). JTN is able to treat mental diseases and help patients to regulate the mind [15]. JTN has been widely used to ameliorate depression and anxiety [16]. A previous study reported that JTN treatment may ameliorate insomnia [17]. We hypothesize that patients receiving JTN will have reductions in drug craving, require less methadone during MMT and have improvements in quality of life, sleep quality, depression and anxiety. Therefore, this study aims to evaluate the efficacy and safety of acupuncture as an adjunctive therapy for patients receiving MMT.

## Methods

### Study design

**Fig. 1:**
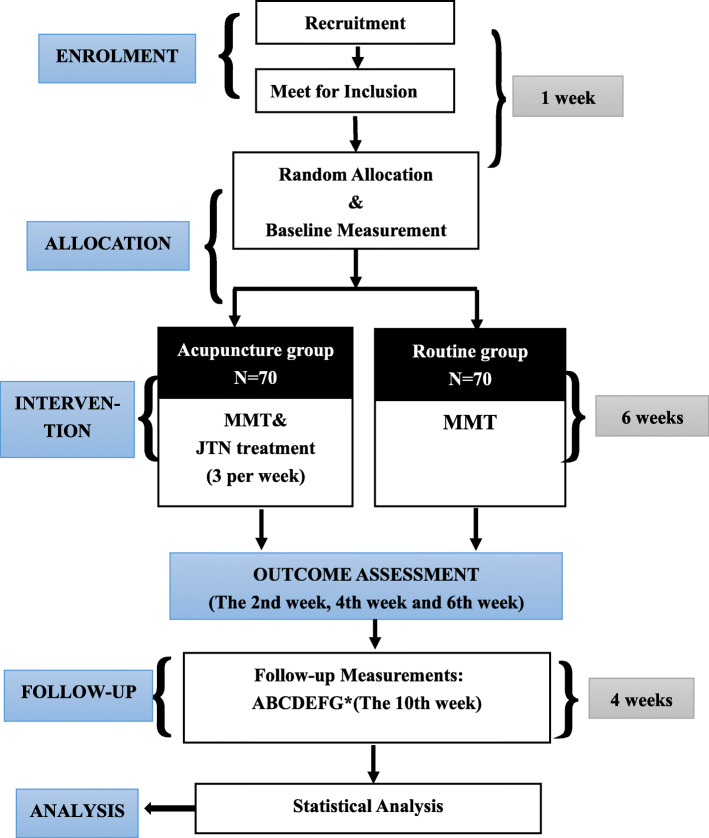
Flowchart of trial procedures

This is a parallel-arm, randomized controlled trial to evaluate the efficacy and safety of acupuncture as an adjunctive therapy for patients receiving MMT. All eligible individuals (140) will be randomly assigned to the acupuncture group (*n* = 70) or the routine group (*n* = 70) in a 1:1 ratio. This study protocol has been approved by the Ethics Committee of the First Affiliated Hospital of Guangzhou University of Chinese Medicine (No.Y-2019-241) and registered in the Chinese Clinical Trial Registry (ChiCTR1900026357). This study protocol conforms to the Standard Protocol Items: Recommendations for Interventional Trials (SPIRIT) guidelines (Additional file 1) [18] and the Standards for Reporting Interventions in Controlled Trials of Acupuncture (STRICTA) checklist (Additional file 2) [19]. Before randomization, all eligible participants will be asked to sign an informed consent. The flowchart of the study is shown in Fig. 1.

### Study subjects

Subjects will be recruited through advertisements at the Affiliated Brain Hospital of Guangzhou Medical University. All eligible individuals will be strictly assessed by an acupuncturist based on the criteria described below. Subjects are eligible to include if they (1) are male or female aged 18–60 years; (2) fulfil the criteria for the diagnosis of opioid dependence on the basis of the Diagnostic and Statistical Manual of Mental Disorders, 5th edition (DSM-V) [20]; (3) receive MMT for more than 30 days; (4) do not receive any kind of acupuncture therapy during the previous 3 months; and (5) are able to sign informed consent.

Subjects will be excluded if they have one or more of the following criteria: (1) serious heart, liver, lung or kidney disease; (2) venereal disease or AIDS; (3) the presence of severe digestive disease and athrepsia; (4) major psychosis; (5) the receipt other treatment that may affect the efficacy evaluation of the present intervention; (6) an infection, inflammation, scar or injury close to the site of the selected acupoints; or (7) are pregnant or planning to become pregnant.

### Randomization, allocation and concealment

Eligible patients will be randomly assigned in an equal ratio to the acupuncture group or the routine group via a central randomization system. The randomization sequence will be generated in a block size of 4. Participants will be informed that they have an equal chance to be assigned to the acupuncture group or the routine group. After participants sign the informed consent form and complete baseline assessments, the researcher will assign them a randomization number and allocate the participants to each group. The allocation of participants will be performed by an independent researcher at the clinical site who was not involved in the assessment of outcomes. Due to the design of this study, practitioners and eligible subjects will know the allocated group, while the outcome assessors, data collectors and statisticians will be blinded to group allocations during the study.

### Intervention

**Fig. 2:**
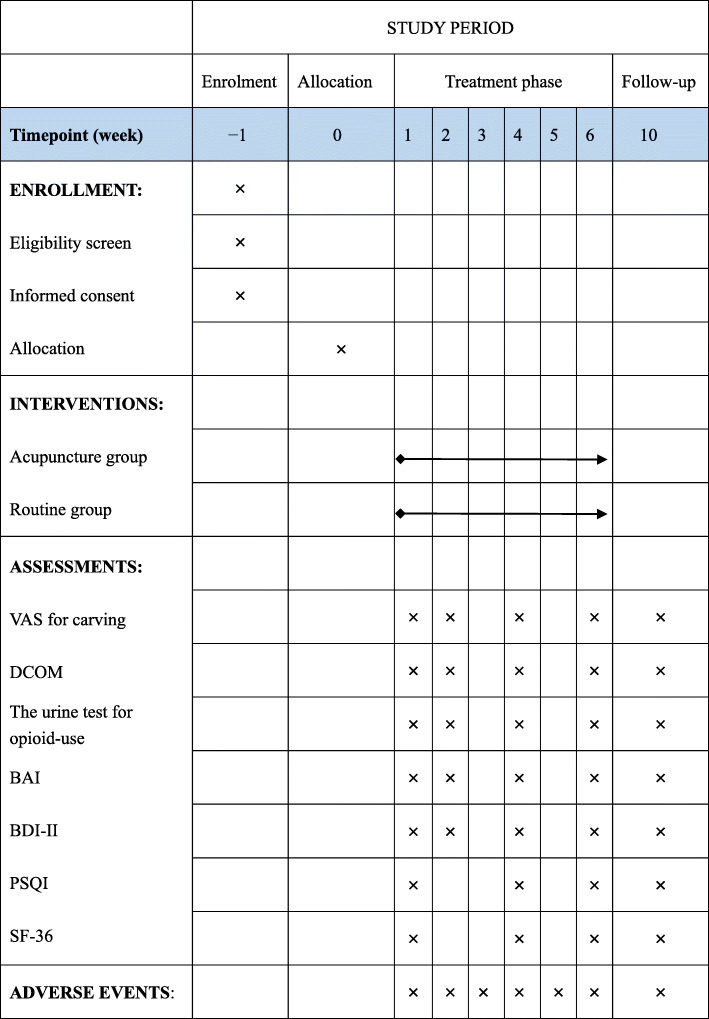
Schedule of enrolment, interventions and assessments

The interventions will be performed by licensed acupuncturists with more than 3 years of experience in clinics. According to the traditional acupuncture theory and previous RCTs, the acupuncture study interventions were developed by a consensus of acupuncture experts. Before initiation of the study, the acupuncturists will obtain special training to gain total understanding of the treatment and receive a brochure that shows the acupuncture manipulation with detailed information. Subjects who are assigned to the acupuncture group will receive MMT and JTN treatment for 30 min per session. Meanwhile, those who are randomized to the routine group will receive MMT only. All 18 sessions of JTN treatment will be delivered over 6 weeks (3 per week) and followed by a 4-week follow-up period. The study schedule is shown in Fig. 2.

#### Acupuncture group

Base on the theory of JTN, every acupoint group has a special name and contains three or four points. In this study, we will choose three acupoints groups, which will be named *Dingshen-zhen*, *Sishen-zhen* and *Shouzhi-zhen*.

**Fig. 3:**
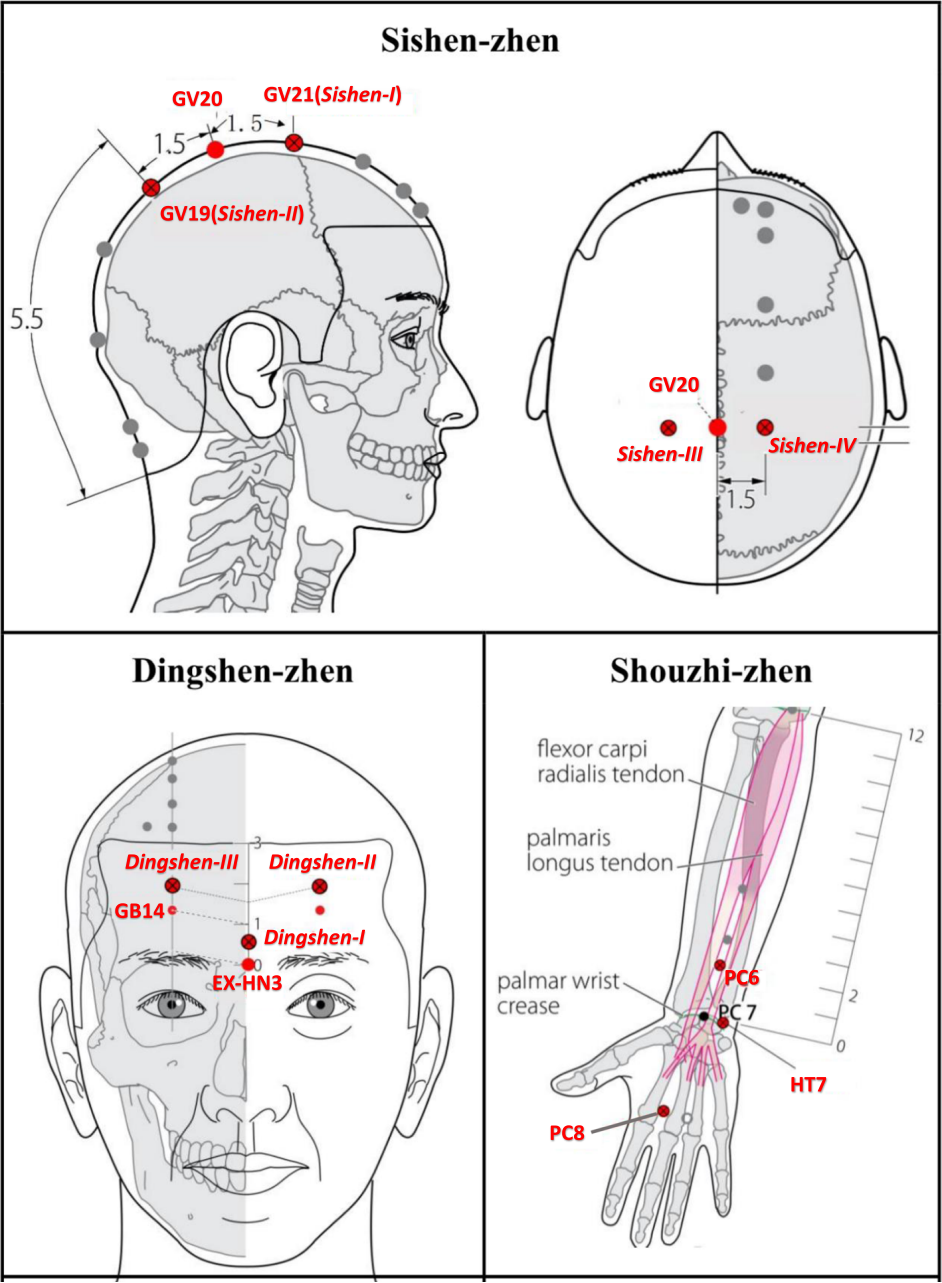
Acupuncture points used in the study

*Dingshen-zhen* will consist of three special points named *Dingshen-I*, *Dingshen-II and Dingshen-III. Dingshen-I* is directly 0.5 *cun* superior to EX-HN3, *Dingshen-II* is directly 0.5 *cun* superior to left GB14 and *Dingshen-III* is directly 0.5 *cun* superior to right GB14. *Sishen-zhen* will consist of two acupoints and two special points named *Sishen-I*, *Sishen-II*, *Sishen-III* and *Sishen-IV*. *Sishen-I* is GV19 and *Sishen-II* are GV21; *Sishen-III* and *Sishen-IV* are, respectively, 1.5 *cun* left and right lateral to the anterior median line and at the same level as GV20. *Shouzhi-zhen* will consist of PC6, PC8 and HT7. The acupuncture points are described in Table 1 and Fig. 3.

**Table 1:** Framework of the acupuncture point prescription

Acupuncture points	Description
GV21: Qianding (*Sishen-I*)	On the head, 3.5 B-cun superior to the anterior hairline, on the anterior median line.
GV19: Houding (*Sishen-II*)	On the head, 5.5 B-cun superior to the posterior hairline, on the posterior median line.
GV20: Baihui	On the head, 5B-cun superior to the anterior hairline, on the anterior median line.
*Sishen-III*	On the head, 1.5*cun* left lateral to the anterior median line and at the same level as GV20.
*Sishen-IV*	On the head, 1.5*cun* right lateral to the anterior median line and at the same level as GV20
EX-HN3:Yintang	On the head, between the right medial end of the eyebrow and the left one.
*Dingshen-I*	On the head, directly 0.5*cun* superior to EX-HN3
GB14: Yangbai	On the head, 1B-cun superior to the eyebrow, directly superior to the centre of the pupil.
*Dingshen-II*	On the head, directly 0.5*cun* superior to left GB14
*Dingshen-III*	On the head, directly 0.5*cun* superior to right GB14
HT7: Shenmen	On the anteromedial aspect of the wrist, radial to the flexor carpi ulnaris tendon, on the palmar wrist crease.
PC6: Neiguan	On the anterior aspect of the forearm, between the tendons of the palmaris longus and the flexor carpi radialis, 2B-cun proximal to the palmar wrist crease.
PC8: Laogong	On the palm of the hand, in the depression between the second and third metacarpal bones, proximal to the metacarpophalangeal joints.

Note: The prescribed acupoints come from the WHO Standard Acupuncture Point Locations in the Western Pacific Region

Sterile stainless-steel disposable acupuncture needles (Huatuo, Suzhou, China; lengths and diameters 0.3 mm × 25 mm or 0.3 mm × 40 mm) will be used in this group. Among the points we choose, *Shouzhi-zhen* will be needled at an angle of 45–90° to the participant’s skin*,* while *Dingshen-zhen* and *Sishen-zhen* will be needled at an angle of 15–30° to the skin. The needles will be inserted at a depth of 5–30 mm. After that, the needles are stimulated manually to achieve the typical acupuncture sensation of *de qi*, which is characterized as soreness, numbness and heaviness. During every session, the acupuncture needles will be retained for 30 min and twirled every 10 min. JTN treatment will be discontinued if the patients suffer from any adverse events, and the acupuncture physicians can decide to terminate the trial.

#### Routine group

All participants in the control group will receive only MMT; they will not receive any treatment related to acupuncture after randomization for 10 weeks. During the study period, there will be five face to face visits and a diary will be collected. After the trial, all participants will have a chance to receive JTN treatment for 6 weeks as compensation.

### Outcome

#### Primary outcome

##### The daily consumption of methadone

Daily consumption of methadone from baseline to the 2nd, 4th, 6th and 10th weeks will be presented. One primary outcome measure will be the change in the daily consumption of methadone from baseline to the 6th week. The enrolled patients will go to the clinic to receive methadone every day, and the dosage will be strictly administered by the doctor, which will be recorded in the clinic computer system.

##### The visual analogue scale for drug craving

The other primary outcome will be the determination of drug craving using a visual analogue scale for drug craving on which the participants will mark on a 100-mm line in proportion to their craving from 0 (no craving) to 100 (strong craving) [21]. The degree of craving will be marked by the subjects at baseline and the 2nd, 4th and 6th weeks after acupuncture administration and at the post-treatment follow-up visit in the 10th week.

#### Secondary outcome

##### The urine test for opioid use

One of the secondary outcome measures will be a positive urine test for opioid use. Subjects will be asked to submit the urine sample at baseline and the 2nd, 4th and 6th weeks after acupuncture administration and at the post-treatment follow-up visit in the 10th week.

##### The 36-item Short Form Survey

The quality of life will be assessed through the Chinese version of the SF-36 from baseline to the 4th, 6th and 10th weeks. Testing of the Chinese version of SF-36 has shown validity similar to that of the original language version [22]. The quality of life is assessed by SF-36 in eight domains: general health (GH), physical functioning (PF), role limitations due to physical problems (RP), role limitations due to emotional problems (RE), bodily pain (BP), social functioning (SF), vitality (VT) and general mental health (MH) [23]. PF, RP, BP, SF and RE will assess the absence of limitations or disability, while the positive state of well-being will be assessed by GH, VT and MH. Subjects who achieve mid-range scores indicate no reported limitations or disabilities [24]. In this study, higher scores will indicate better health status.

##### Pittsburgh sleep quality index (PSQI)

Sleep quality will be assessed using the Chinese version of the PSQI [25] from baseline to the 4th, 6th and 10th weeks. The Chinese version of the PSQI has demonstrated reliability and validity similar to that of the original language version [26]. The PSQI evaluates sleep disturbances through subjective sleep quality, sleep duration, sleep latency, habitual sleep efficiency, sleep disturbances, daytime dysfunction and use of sleeping medication. Each item is graded on 4-point scale (0–3), which is summed together to gain a total score (0 to 21). Higher scores show that patients suffer a higher severity of sleep disturbance, and a total score higher than 5 indicates “poor sleep”.

##### The Beck Depression Inventory-II (BDI-II)

BDI-II, a self-reported questionnaire, will be used to assess the severity of depression, which consists of 21 symptoms [27]. Each symptom is graded on 4-point scale (0–3), which is summed together to yield a total score (0 to 63). Higher scores mean a greater severity of depression. The Chinese version has been validated in adolescents [28]. The BDI-II will be assessed at baseline and the 2nd, 4th, 6th and 10th weeks.

##### The Beck Anxiety Inventory (BAI)

Anxiety symptoms will be assessed by the BAI, which is a 21-item questionnaire [29]. Each symptom is rated on a 4-point scale (0–3). The maximum score is 63, and higher scores indicate greater severity of anxiety. The BAI will be assessed at baseline and at the 2nd, 4th, 6th and 10th weeks.

### Safety

All participants will have some tests to check their body at screening and after 6 weeks of treatment. These tests will be relative to the heart, liver, kidney and other organs, including white blood cell count, haematocrit, haemoglobin, platelets, aspartate aminotransferase/alanine aminotransferase, blood urea nitrogen, creatinine, gamma-glutamyl transpeptidase, erythrocyte sedimentation rate and electrocardiogram.

Adverse events (AEs) are defined as any unexpected unfavourable signs, symptoms or feelings occurring in subjects during the entire period, whether or not associated with JTN treatment. Every AE will be fully recorded on the case report form (CRF), including all details of AEs. Serious AEs that threaten a participant’s life or result in hospitalization will be reported to the Research Ethics Committee concerned within 24 h. All the tests and AEs are recorded, with an aim to evaluate the safety of JTN.

### Quality control, data management and monitoring

Before recruitment, the entire team will be required to attend a training workshop, which ensures their strictly compliance with the study protocol and total understanding of the trial administration process. The interventions will be performed by licensed acupuncturists with more than 3 years of experience in hospitals or clinics. They will also receive a brochure about the protocol and standard operating procedures of JTN.

The data will be carefully collected and recorded on CRFs. All data will be put onto a computer with password protection by staff blinded to the group allocation and checked twice by investigators after data entry. Data quality will be checked regularly by research assistants and supervised by monitors. The original CRFs and all other forms will be archived securely at the Medical College of Acu-Moxi and Rehabilitation of Guangzhou University of Chinese Medicine. The South China Research Center of Guangzhou University of Chinese Medicine will be responsible for monitoring for the data, regulatory management of this trial, auditing the trial every 3 months independently of investigators and the sponsor and deciding on any premature closure of the study.

### Sample size calculation

The primary outcome measure for drug craving is VAS. Based on previous studies [5, 30], after 4 weeks of treatment, the VAS for drug craving score difference between the baseline and the endpoint in the sham acupuncture group should be approximately 20 ± 30, while the true acupuncture group should be approximately 37.5 ± 28. Type 1 error is assumed at 0.05, and type 2 error is assumed at 0.1. PASS11.0 software (CSS Statistical Software, Kaysville, UT, USA) has been used to determine the sample size, and the minimum sample size is 60 subjects for each group. Considering a dropout rate of 15%, a total of 140 subjects will be required with 1:1 allocation to each group (70 participants per group) for this study.

### Statistical analysis

All analyses will be conducted based on the intention-to-treat principle and per-protocol (PP) analysis. Missing data will be replaced according to the principle of multiple imputations. Descriptive statistics will be presented for each group as the mean change (SD, 95% confidence intervals). All statistical tests will be analysed by using the SPSS statistical software version 20 (IBM SPSS Statistics, New York, USA). *P* values < 0.05 (two-sided) are considered statistically significant. Demographic and baseline data will be tabulated and evaluated using analysis of variance (ANOVA) or the *χ*^2^ test. To compare means between two groups, we will use the independent sample *t* test. Two-way repeated-measures analysis of variance (ANOVA) will be carried out to explore time × treatment interaction (“time” is the unit of measurement).

## Discussion

To our knowledge, JTN treatment for patients receiving MMT has not been intensively investigated. This study describes the protocol of a randomized controlled trial to test the effectiveness and safety of acupuncture as an adjunctive therapy for patients receiving MMT.

JTN, a type of acupuncture technique, has been practised for decades. One of the features of JTN is choosing treatment acupoints in a unique way. The prescription of JTN is comprised of several acupoint groups, and each acupoint group consists of three or four special acupoints that are based on local points, influential points or empirical points. The law of combination of these points may have more effectiveness to treat disease than a normal mixture of ‘cookbook’ points.

According to previous studies, the effectiveness of acupuncture as an adjunct therapy in patients receiving MMT has been controversial. Bearn reported a lack of effect for adjunctive MMT with auricular acupuncture upon withdrawal symptoms or drug craving [31]. Pei Lin showed a lack of effect for adjunctive MMT with auricular acupuncture upon the number of daily consumed cigarettes, withdrawal syndrome and relapse rate [32]. However, several studies have reported positive findings in the effectiveness of acupuncture for the treatment of drug dependence [33–35]. Chan claimed that 4 weeks of acupuncture as an adjunct to MMT may reduce the daily dosage of methadone and may cause a greater improvement in sleep latency [5]. Although JTN has been practised for several decades, this is the first time that JTN will be applied to treat opioid dependence. According to the theory of TCM, the acupoints we choose may help to tranquilize and free the body from tension, which may have the potential to ameliorate several mental or physical symptoms of opioid dependence.

One important limitation of our study is that it is an open-label trial, making it impossible to execute a blinding method. Patients may have strong treatment preferences for acupuncture, which may produce adjuvant treatment effects. To reduce this kind of bias, the acupuncturists and outcome assessors will be different, which will maintain the objectivity of assessment of outcomes. We will exclude the investigators and statisticians from randomization as well, to make sure all of them are blind to allocation.

In conclusion, the results of this trial will provide evidence on the effectiveness and safety of acupuncture as adjunctive therapy for patients receiving MMT.

## Trial status

This trial is currently recruiting patients, which started on 5 October 2019 and is anticipated to end on 1 December 2020. The protocol is Version 3.0 dated 2019/09/16.

## Supplementary information

**Additional file 1.** Standard Protocol Items: Recommendations for Interventional Trials (SPIRIT) Checklist.

**Additional file 2.** Standards for Reporting Interventions in Clinical Trials of Acupuncture (STRICTA).

## Data Availability

The full protocol of this study will be provided by the corresponding author. Datasets generated or analysed during the current study will be available from the corresponding author on reasonable request.

## Abbreviations

JTN: Jin’s three-needle acupuncture
TCM: Traditional Chinese medicine
DSM-V: Diagnostic and Statistical Manual of Mental Disorders, 5th edition
PSQI: Pittsburgh sleep quality index
BDI-II: Beck Depression Inventory-II
BAI: Beck Anxiety Inventory
DCOM: Daily consumption of methadone
MMT: Methadone maintenance therapy
AEs: Adverse events
CRFs: Case report forms
PP: Per-protocol
