# Supplementary material for: Effect of acupuncture for methadone maintenance treatment patients: study protocol of a randomized clinical trial
Source: Trials. 2020 Dec 7;21:1003. doi: 10.1186/s13063-020-04930-x (PMC7720473; doi:10.1186/s13063-020-04930-x)
Supplement: Supplementary file 2 — Additional file 2. Standards for Reporting Interventions in Clinical Trials of Acupuncture (STRICTA). [file 13063_2020_4930_MOESM2_ESM.docx]

Table S2. Standards for Reporting Interventions in Clinical Trials of Acupuncture( STRICTA )

| **Section/topic** | **Item number** | | | **Checklist item** | Report/  Not |
| --- | --- | --- | --- | --- | --- |
| 1.Acupuncture rationale | | | | | |
|  | | 1a | Style of acupuncture (eg, Traditional Chinese Medicine, Japanese, Korean, Western medical, Five Element, ear acupuncture, etc) . | | P4-5 |
|  |  | 1b | Reasoning for treatment provided, based on historical context, literature sources and/or consensus methods, with references where appropriate . | | P4-5 |
|  |  | 1c | Extent to which treatment was varied . P7-8 | | |
| 2.Details of needling | |  |  | |  |
|  | | 2a | Number of needle insertions per subject per session (mean and range where relevant) | | Table 1, Figure 3 |
|  |  | 2b | Names (or location if no standard name) of points used (uni-/bilateral) . | | Table1, Figure 3 |
|  | | 2c | Depth of insertion, based on a specified unit of measurement or on a particular tissue level . | | P7-9 |
|  |  | 2d | Responses sought (eg, *de qi* or muscle twitch response) . | | P7-9 |
|  | | 2e | Needle stimulation (eg, manual or electrical) . | | P8 |
|  |  | 2f | Needle retention time . | | P8 |
|  | | 2g | Needle type (diameter, length and manufacturer or material) . | | P8 |
| 3.Treatment regimen | |  |  | |  |
|  | | 3a | Number of treatment sessions . | | P7 |
|  |  | 3b | Frequency and duration of treatment sessions . | | P7 |
| 4.Other components of  treatment | |  |  | |  |
|  | | 4a | Details of other interventions administered to the acupuncture group (eg, moxibustion, cupping, herbs, exercises, lifestyle advice) . | | P7-8 |
|  |  | 4b | Setting and context of treatment, including instructions to practitioners, and information and explanations to patients . | | P7 |
| 5.Practitioner  background | |  |  | |  |
|  | | 5 | Description of participating acupuncturists (qualification or professional affiliation, years in acupuncture practice, other relevant experience) . | | P7 |
| 6.Control or comparator  interventions | |  |  | |  |
|  | | 6a | Rationale for the control or comparator in the context of the research question, with sources that justify the choice(s). | | P4,7 |
|  | | 6b | Precise description of the control or comparator. If sham acupuncture or any other type of acupuncture-like control is used, provide details as for items 1-3 above. | | P9 |

This checklist should be read in conjunction with the explanations of the Standards for Reporting Interventions in Clinical Trials of Acupuncture items.
